# The Neuron Regrowth Is Associated with the Proliferation of Neural Precursor Cells after Leukemia Inhibitory Factor Administration following Spinal Cord Injury in Mice

**DOI:** 10.1371/journal.pone.0116031

**Published:** 2014-12-26

**Authors:** Yubo Li, Dawei Zang

**Affiliations:** 1 Capital Medical University, Beijing, 100069, China; 2 Department of Neurology, Tianjin First Center Hospital, Tianjin Medical University, Tianjin, 300192, China; Baylor College of Medicine, United States of America

## Abstract

**Objectives:**

To explore whether LIF could promote the proliferation of neural precursor cells (NPCs) and to analyze the correlation between increased NPCs and FluoroGold (FG) labeled neurons in mice after spinal cord injury (SCI).

**Methods:**

Motor behavior was assessed using Rotarod and Platform Hang tests; neurons in the corticospinal and rubrospinal systems were labeled with FG, NPCs were immustained with nestin-FITC conjugate. The numbers of FG-labeled neurons and NPCs were estimated, and the correlation between FG-labeled neurons and NPCs was assessed.

**Results:**

Mice in the SCI group showed negligible recovery of locomotor behavior; in contrast, mice in the LIF group showed a statically significant improvement. Both FG-labeled neurons and NPCs were significantly increased in the LIF group compared to the SCI group, and this increase in FG-labeled neurons and NPCs showed a clear association above the lesion level.

**Conclusions:**

LIF could promote locomotive behaviors in mice post-SCI by encouraging the proliferation of NPCs; LIF may in fact be a potential cytokine for the induction of NPCs post-SCI.

## Introduction

The functional loss after spinal cord injury (SCI) is mainly associated with the irreversible damage of neuron and axon in the spinal cord; the mechanisms underlying functional recovery after injury remain only partially understood. Although many studies leading to potential therapies for SCI have been conducted over the past few decades [1] [2] [3] [4], there is to date no satisfactory treatment option for SCI patients. Many studies have focused on the transplantation of stem cells into the central nervous system (CNS) [5] [6] [7], [8]; however, some other studies have found that endogenous neural precursor cells (NPCs) may in fact be another potential method for function recovery after SCI [9] [10] [11] [12].

NPCs have been detected in the CNS of normal mice in many zones, such as olfactory bulb core, hypothalamus, ependymal zones, around the central canal of the spinal cord, throughout the parenchyma of spinal cord, and midline structures of brain and spinal cord [13]. These NPCs are activated in some disease conditions, such as neurodegenerative diseases, stroke and brain and spinal cord injury and may function to recover lost neuro-function by remodeling the milieu interne of the lesion zone [14] [15]. To date, we have not adequately paid attention to the induction and recruitment of endogenous NPCs in the application of treatment for SCI.

We previously reported that leukemia inhibitory factor (LIF) could promote the recovery of locomotor function following SCI [16]; LIF is a well-known neuroprotective cytokine in SCI. Of particular relevance to this study is the recently recognized effect of LIF in preventing oligodendrocyte death and demyelination in SCI, and in encouraging the proliferation of NPCs in some disease conditions [17] [18] [19]. Furthermore, LIF can protect axons and increase corticospinal axonal regrowth in animal SCI models.

LIF can promote motor function and increase the proliferation of NPCs [20] [21], which may contribute to the recovery of neurological function after SCI. No study to date has examined endogenous NPCs within the context of SCI after LIF administration. A clear understanding of this functional recovery related to the correlation between the increase of motor neurons and the proliferation of NPCs can provide future strategies for harnessing NPCs in therapies for SCI. In this study, we aimed to reveal the patterns of neuron regrowth in the corticospinal and rubrospinal systems and the proliferation of NPCs after LIF administration, and we assessed the correlation between neuron regrowth and NPCs proliferation after LIF administration in the SCI condition.

## Material and Methods

C57BL/6J mice aged eight weeks were used in this experiment. The Animal Ethics and Experimentation Committee of Tianjin First Center Hospital approved all experimental procedures performed in this study (YZXA11201). Both male and female mice were studied, and because no gender-linked difference was found, the data from both genders were pooled. Table 1 showed the mice allocated to this study. The SCI procedure was described in detail previously [16], briefly, mice were anesthetized with ketamine (0.1 mg/g IP) and ilium xylazine anesthesia (0.014 mg/g IP). A skin incision of approximately 1.0 cm in length was made, followed by a laminectomy to expose the T_12_ spinal cord segment, and the lesioning was performed using iridectomy scissors and approximately two-thirds of the spinal cord was cut off. This lesioning procedure resulted in consistent transaction of the most rubrospinal and dorsal and ventral corticospinal tracts ipsilaterally at T_12_, all mice with SCI were able to drink, eat independently, urinate and defecate after recovering from anesthesia. At 7th day post lesion, a laminectomy was carried out again at the L_1_ segment and a small piece of sterile gelatin sponge (Gelfoam) soaked with 0.2 ml of 4% FluoroGold (FG) (Fluorochrome, Denver, CO) was inserted into the incision, to a depth of approximately 1 mm. The incisions through muscles, connective tissues and skin were separately closed using surgical sutures. Mice in Normal group (Nor) received the same surgical procedure except spinal cord injury, and mice in Sham group received the same surgical procedure except spinal cord injury and FG insertion.

**Table 1:** Experimental design showing the number of mice allocated to the Nor, SCI, LIF and Sham groups.

	Nor	SCI	LIF	Sham
**Male**	6	6	6	6
**Female**	6	6	6	6

Mice in the LIF and SCI groups received daily intraperitoneal injections of LIF (25 µg/kg) and VEH (1% albumin in 0.1 M mouse tonicity phosphate-buffered saline), treatments were initiated when the animals had regained consciousness 2 h after the completion of the SCI in the two groups.

All mice were assessed every second day using Rotarod and Platform Hang tests, these two tests were described in detail previously [16], briefly, (a) for the *Rotarod test*, performance was defined as the time period in which each mouse remained on the rotating axle (3.6 cm diameter; speed of rotation, 16 rpm) for up to a maximum of 180 seconds without falling. A mean value from three trials was then obtained; (b) the *Platform hang test*, coordinated motor function, the ability of the paralytic hind limb to assist the mouse in climbing to the top of a transparent plastic plate when its forelimbs were placed on a plate edge and the animal was suspended above soft bedding. The distance that the paralytic right hind limb moved to the top of the plate was recorded.

At 14^th^ day post lesion, the mice were killed by a lethal intraperitoneal injection of 200 mg/kg of sodium pentobarbitone (Rhone Merieux, Australia) and perfused transcardially with 0.1 M mouse tonicity phosphate buffered saline (MTPBS), pH 7.4, followed by 4% (w/v) paraformaldehyde (4% PFA). The procedure of tissue processing was described in our previous study [16], briefly, the brain, brainstem and spinal cord were dissected and postfixed in 30% sucrose overnight at 4°C. Then samples were sectioned coronally at 10 µm using a Cryostat, and every 10th section was collected. For FG-labeled neuron counting, the sections were air-dried, immersed in xylene and cover-slipped with non-fluorescent DPX plastic mounting media. The number of FG-labeled neurons was counted at ×10 using a grid in the eyepiece to avoid double counting and expressed as the mean±Se. For nestin immunostaining, six parallel series from each animal were mounted on super frost coated slides and immunostained for nestin immunostaining.

For nestin immunostaining, briefly, we directly conjugated our nestin mouse monoclonal antibody to FITC using the monoclonal antibody labeling kit (Alexa Fluor 488, Molecular probes, U.S.A.). The procedure of nestin-FITC conjugate was described in our previous study [22], briefly, 100 µl of 1 mg/ml anti-nestin monoclonal antibody (MAB353, Chemicon, California, U.S.A.) was added 10 µl of 1 M sodium bicarbonate, this was transferred to a vial of Alexa Fluor 488 reactive dye and incubated for 1 hour at room temperature, the labeled anti-nestin antibody was then purified using spin columns according to the manufacturer's instructions, the absorbance of the purified antibody was measured at both 280 nm and 494 nm to determine the protein concentration (M) and the moles of dye bound per mole of antibody, nestin-FITC conjugate then was diluted 50-fold with antibody buffer.

The sections were washed three times in MTPBS for 20 minutes and incubated with the nestin-FITC conjugate (diluted 1∶50) overnight at 4°C. Then the sections were rewashed three times in MTPBS and cover-slipped using fluorescent mounting media (DAKO, California, U.S.A.), the number of NPCs from 3 mm in length of above and below the lesion level was counted under fluorescence microscopy using green (nestin) color illumination at a magnification of 20× with a grid.

### Statistical Analysis

An unpaired *t*-test and one-way analysis of variance (ANOVA) with Tukey's posthoc test using GraphPad Prism (Version 4.0, GraphPad Software Inc., San Diego, USA) were performed in this study. Pearson's correlation test was used for statistical analysis of correlations between the number of NPCs and FG-labeled neurons. Statistical data were expressed as the mean±Se, statistical significance was set at p-values below 0.05.

## Results

**Figure 1:**
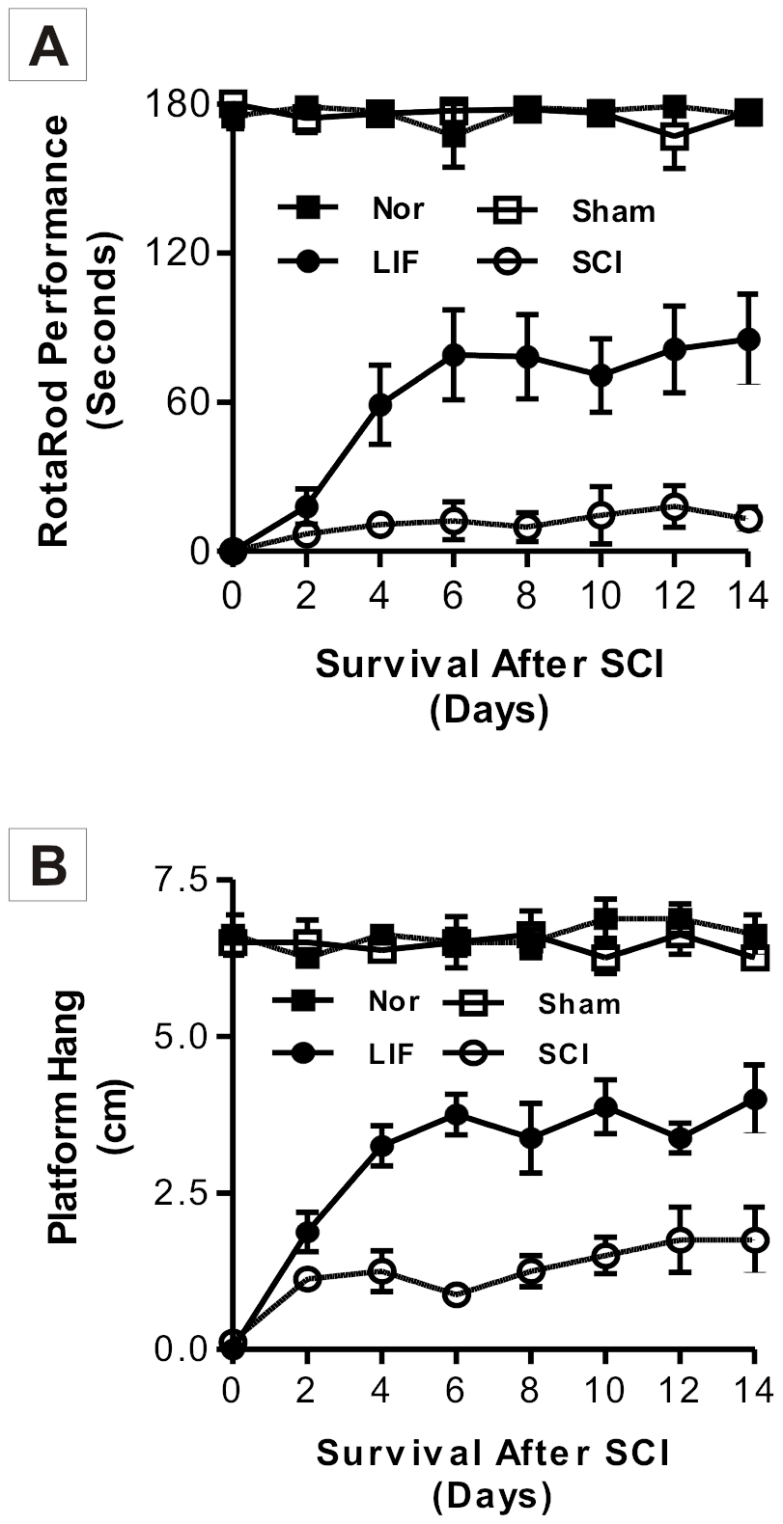
Locomotor behavior analysis of mice in the Nor, SCI, LIF and sham groups using the Rotarod and the Platform Hang tests. Fig. 1A showed a permanent hind limb paralysis in Rotarod analysis in the SCI group. A significant improvement was detected on the Rotarod in the LIF group compared to the SCI group; a statistical difference was found after the fourth day and the maximal response was achieved by the sixth day, despite continuous treatment for 14 days. A similar significant (*p<*0.05) improvement was observed in the Platform Hang test (Fig. 1B) in LIF group. The locomotor behavior of the Nor and Sham groups was not affected in both Rotarod and Platform Hang tests.

The first part of this study involved the analysis of locomotor function (Fig. 1), Fig. 1A-B showed locomotor behavior of mice in the Rotarod and Platform Hang tests. Fig. 1A showed that mice in the Nor and Sham groups could balance on the Rotarod for 180 seconds, and although there was locomotor improvement in the second day of the LIF group, this difference was not statistically significant (p>0.05) compared to the SCI group. From 4^th^ day following SCI, mice in the LIF group (69.38±15.85) showed a significant improvement compared to the SCI group (4.87±1.34), and this improvement achieved its maximum by day six despite continuous treatment for fourteen days. The same pattern was also observed in the Platform Hang test. The recovery of locomotor function was statistically significant in LIF group (2.81±0.52 cm) compared to the SCI group (1.44±0.22 cm) from the 4^th^ day following SCI (Fig. 1B).

**Figure 2:**
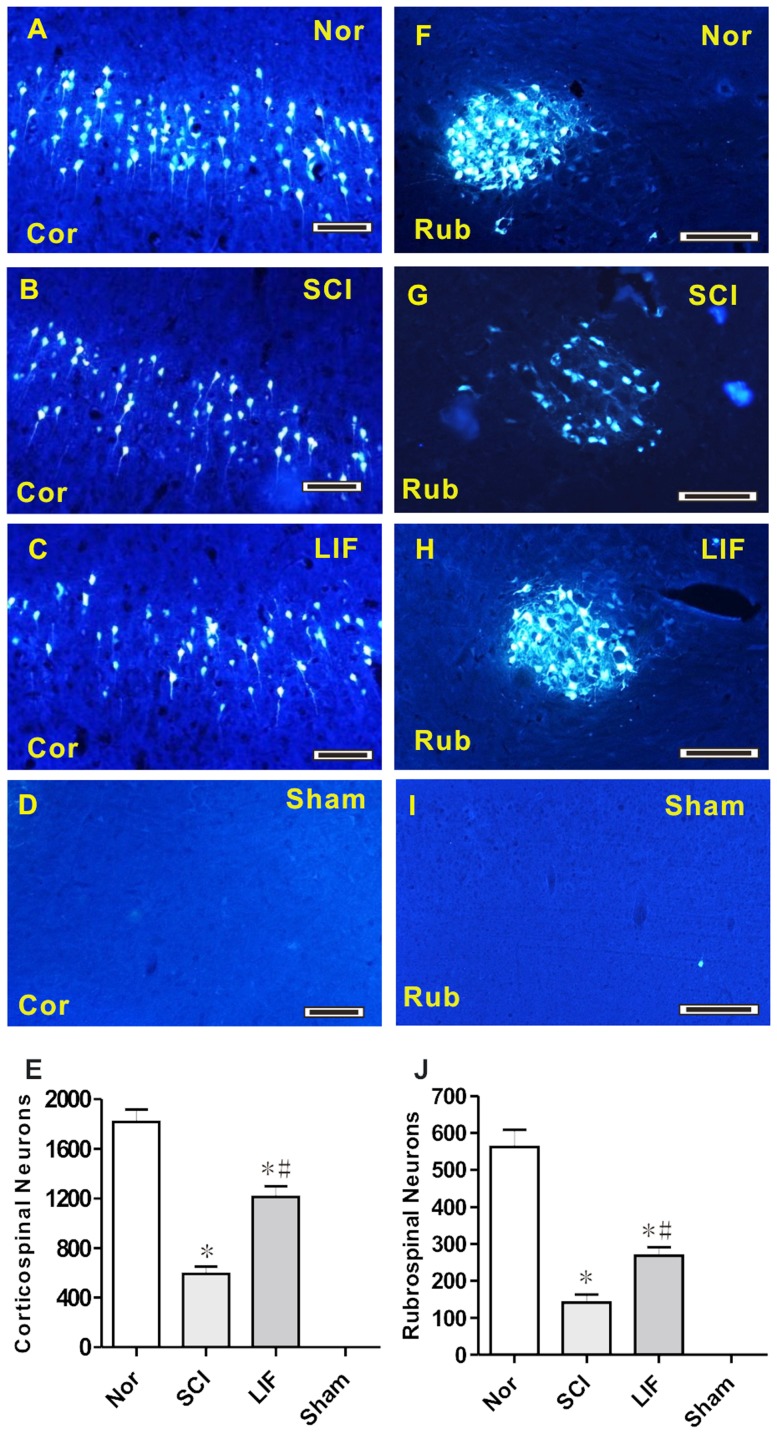
Analysis of FG-labeled neurons in the corticospinal and rubrospinal systems in the Nor, SCI, LIF and Sham groups. Fig. 2A–D showed the FG-labeled neurons in the corticospinal system in the Nor (2A), SCI (2B), LIF (2C) and Sham (2D) groups. Fig. 2E summarized the number of FG-labeled neurons in the corticospinal system. The number of FG-labeled neurons in the LIF group decreased compared to the Nor group, but increased compared to SCI group. Fig. 2F–I showed the FG-labeled neurons in the rubrospinal system in the Nor (2F), SCI (2G), LIF (2H) and Sham (2I) groups. Fig. 2J showed a similar pattern to that shown in Fig. 2E in the rubrospinal system. * Indicates comparison with the Nor group. # Indicates comparison with the SCI group. Cor: Corticospinal; Rub: Rubrospinal. Bar =  100 µm.

The second part of this study involved the quantification of FG-labeled neurons in corticospinal and rubrospinal systems in SCI and LIF treated mice following SCI (Fig. 2). In corticospinal system, FG-labeled neurons were significantly decreased in both SCI (2B) and LIF (2C) groups compared to Nor group (2A), and no FG-labeled neurons were detected in the Sham group (2D). Histogram 2E shows that FG-labeled neurons in LIF group decreased compared to the Nor group, but markedly increased compared to the SCI group. In the rubrospinal system, FG-labeled neurons were significantly decreased in both the SCI (2G) and the LIF (2H) groups compared to the Nor group (2F), and no FG-labeled neurons were detected in the Sham group (2I). Histogram 2J shows that FG-labeled neurons in LIF group decreased compared to Nor group, but markedly increased compared to the SCI group.

**Figure 3:**
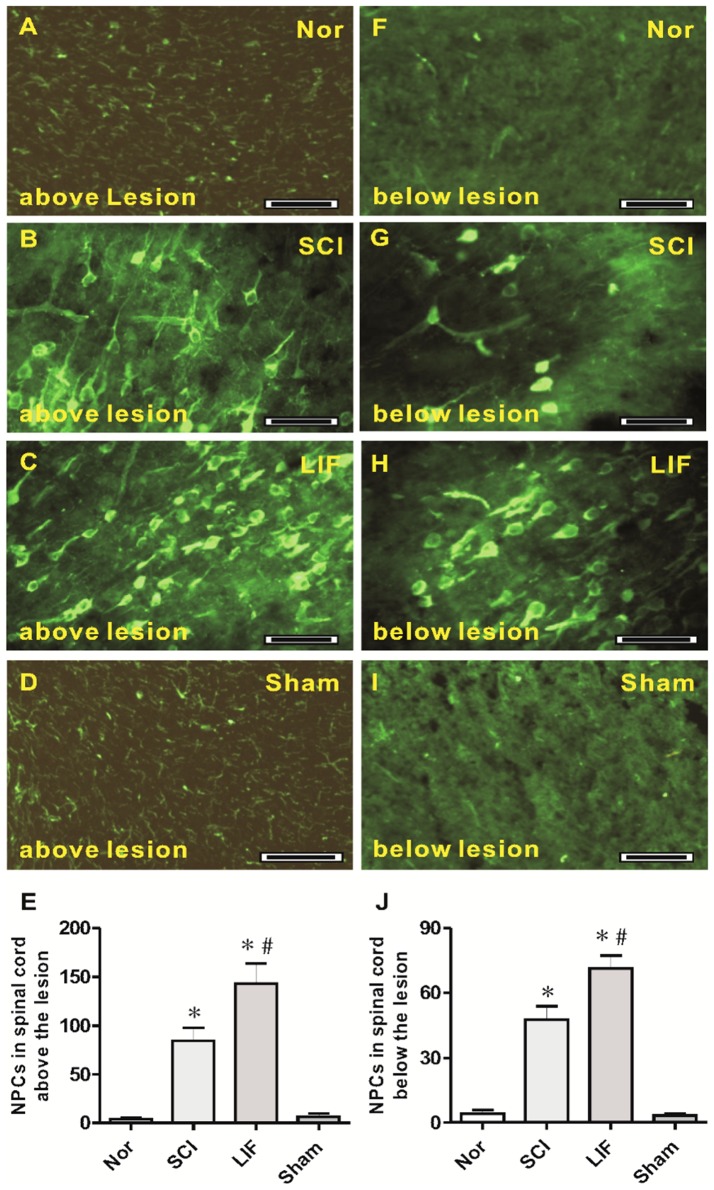
NPCs in spinal cord above and below the lesion level. Fig. 3A–E showed the NPCs in spinal cord above the lesion level. Fig. 3A–D showed the NPCs above the lesion level in the Nor (3A), SCI (3B), LIF (3C) and Sham (3D) groups. Fig. 3E showed that the number of NPCs above the lesion level in the LIF group increased compared to the SCI group, but they were rare in the Nor and Sham groups. Fig. 3F–J showed the NPCs in the spinal cord below the lesion level. Fig. 3F–I showed the NPCs below the lesion level in the Nor (3F), SCI (3G), LIF (3H) and Sham (3I) groups. Fig. 3J showed the number of NPCs below the lesion level in LIF group increased compared to the SCI group, but they were rare in the Nor and Sham groups. * indicates comparison with the Nor and Sham groups. # Indicates comparison with the SCI group. Bar  = 50 µm.

The third part of this study involved the quantification of NPCs above and below the lesion level of the spinal cord in SCI- and LIF-treated mice following SCI (Fig. 3). At the level above the lesion, a number of NPCs were observed in both the LIF (3C) and the SCI (3B) groups, but were rarely seen in the Nor (3A) and Sham (3D) groups. Histogram 3E showed a marked increase in NPCs in the SCI (84.42±13.31) and LIF (143.22±20.68) groups. The number of NPCs in the LIF group was much higher than that in SCI group. At the level below lesion, some NPCs were also observed in both the LIF (3H) and the SCI (3G) groups, but were rarely seen in the Nor (3F) and Sham (3I) groups. Histogram 3J showed a marked increase in NPCs in the SCI (47.58±6.23) and the LIF (71.33±5.89) groups, and the number of NPCs in the LIF group was higher than that in the SCI group below the lesion level.

**Figure 4:**
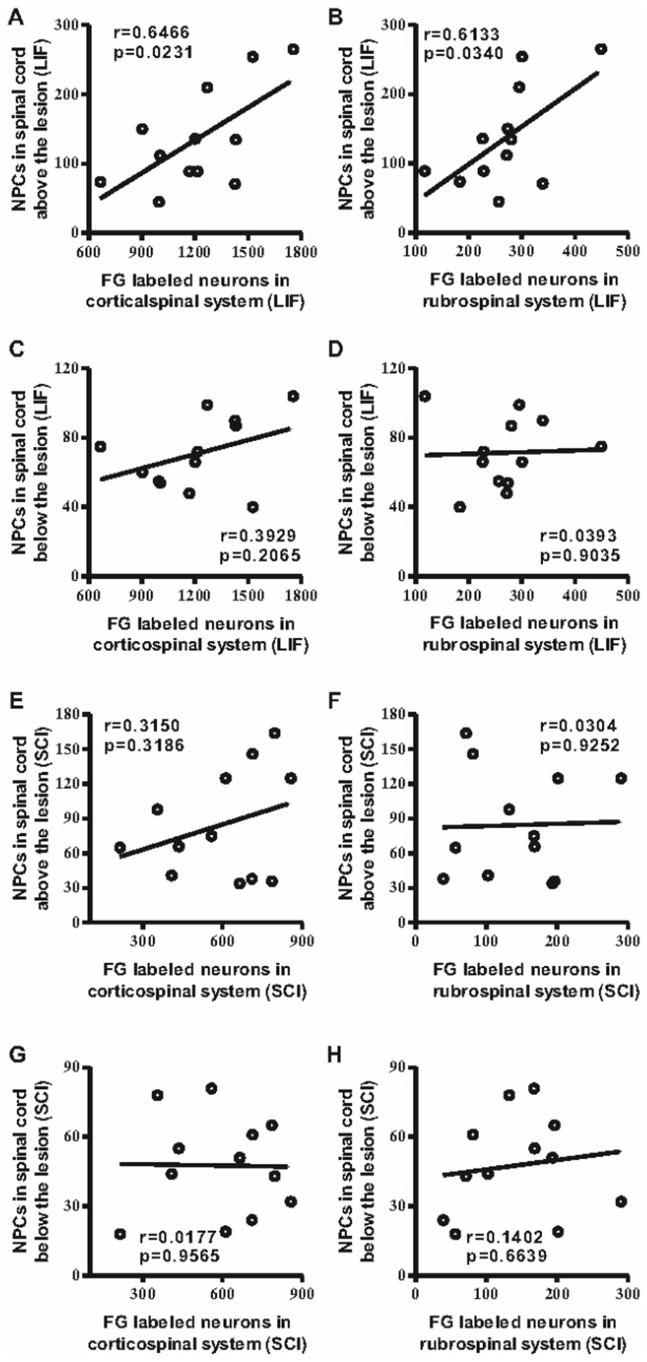
The correlation between NPCs and FG-labeled neurons. Fig. 4A–B showed that the number of NPCs above the lesion level, but not below the lesion level (Fig. 4C–D), was positively correlated with the number of FG-labeled neurons in both the corticospinal and rubrospinal systems in the LIF group. There was no any positive correlation detected between the number of NPCs and the number of FG-labeled neurons above (Fig. 4E–F) and below (Fig. 4G–H) the lesion level of spinal cord in both the corticospinal and rubrospinal systems in the SCI group.

The fourth part of this study involved a correlation analysis between the number of NPCs and FG-labeled neurons above and below the lesion level in LIF and SCI groups (Fig. 4). The increase of NPCs above the lesion level in LIF group was significantly correlated with the increase of FG-labeled neurons in either corticospinal (4A) or rubrospinal (4B) systems, but no correlation was detected below the lesion level in the LIF group (4C–D). For the SCI group, we did not find any significant correlation between the numbers of NPCs and FG-labeled neurons above (4E–F) and below (4G–H) the lesion level in either the corticospinal or the rubrospinal systems.

## Discussion

To investigate functional recovery after SCI, a major requirement was to develop a reliable SCI mouse model; we used a previously described animal model that produced unilateral limb paralysis without concurrent loss of bladder and bowel control [16]. After SCI, the long-term deficit of motor function in mice was verified by two tests (Rotarod and Platform Hang). In this mouse model, the corticospinal and rubrospinal tracts were unilaterally completed cut off in the right side, and the left side remained untouched.

In this study, we used this SCI model to assess LIF efficacy. The motor function in the LIF treated group increased significantly, and the number of FG-labeled neurons in both the corticospinal and the rubrospinal systems also markedly increased in the LIF group compared to the number in the SCI group. We postulated the recovery of motor function and the increase of FG-labeled neurons were due to the regrowth and remylination of axons from both the corticospinal and the rubrospinal tracts. We supposed that LIF plays a key role in preventing oligodendrocyte apoptosis, promoting the regrowth and synapses of axons after lesion. Several previous studies support our findings. Endogenous LIF significantly reduces inflammatory axonal loss and improves neurological outcomes [23], and Bradley reported that LIF promotes oligodendrocyte survival after SCI by augmenting the expression of insulin-like growth factor 1 (IGF–1) [19]. LIF reduces oligodendrocyte apoptosis, prevents the secondary wave of demyelination and decreases inhibitory myelin deposits by inducing the signaling pathways of JAK/STAT and Akt and promoting the expression of the antiapoptotic molecule factor, cIAP2 [24] [25]. LIF-secreting fibroblasts also promote axonal sprouting of the corticospinal tracts after SCI [26]. It should be noted that LIF penetrates into the damaged spinal cord due to the disrupted blood-spinal cord barrier; meanwhile, it has also been confirmed that LIF readily crosses the blood-spinal cord barrier by specific upregulation of transport involving LIFRa receptor [27] [28].

This study also showed that NPCs were clearly detected below and above the lesion level in SCI mice. This finding suggests that NPCs were reactivated post SCI and closely associated with the course of pathology and the attempt of regeneration. A number of studies have reported this NPC response in different disease conditions, NPCs in a mouse model of ALS were reactivated at disease onset and markedly increased with disease development, NPCs mainly differentiated into astrocytes, but a small percentage of NPCs differentiated into neurons and oligodendrocytes [29] [30]. However, high percentages of astrocytes are adverse to remyelination, enhancing the plasticity of sensory axons, and producing allodynia [31] [32] [33]. NPCs could also improve cognitive function in Alzheimer's disease [34] [35].

NPCs in the LIF-treated group significantly increased compared to the SCI group both above and below lesion level, and a high number of NPCs were found above the lesion level compared to below. This finding further confirmed the neuroprotective effects of LIF in disease conditions. It has been reported that NPCs are enhanced by systemic administration of LIF in a PD animal model [36]. LIF promotes NPCs migration *in vitro*, and LIF can stimulate the proliferation of olfactory NPCs by promoting iNOS expression and enhancing nitric oxide level. On the other hand, it has been confirmed that NPCs could secrete endogenous LIF to enhance the survival, differentiation and the remyelination capacity of both endogenous oligodendrocyte precursors and mature oligodendrocytes [37] [38] [39].

In the present study, we found that LIF can ameliorate motor function and enhance the proliferation of NPCs, and we postulated that the regrowth of FG-labeled neurons, which contributes to the functional recovery, might in fact be associated with this increase in NPCs. Therefore, we analyzed the correlation between the number of FG-labeled neurons in the corticospinal and rubrospinal systems and the number of NPCs above and below the lesion level. We found NPCs above the lesion level significantly correlated with FG-labeled neurons in both the corticospinal and rubrospinal systems in the LIF-treated group, but not in the SCI group. There was no any association detected below the lesion level. These results underscored that there is a correlation between NPCs above (but not below) the lesion level in the spinal cord and neuron regrowth in brain. These reactivated NPCs, which contribute to the recovery, are mainly located above the lesion level. Furthermore, because of factors such as LIF, they could promote the action of NPCs above the lesion level. This finding also suggests that induction-using factors inherent to injury mechanisms are required for the proliferation of NPCs in the spinal cord after SCI.

In summary, we demonstrated that the NPC response was initiated after SCI, but the limited number of NPCs, the action of inhibitory factors and the insufficient growth factors for oligodendrocytes survival and remyelination in the spinal cord may all lead to the failure of functional recovery. However, we found that LIF plays a key role in promoting the regrowth of neurons and encouraging the proliferation of NPCs. This neuron regrowth is consistent with NPC proliferation after LIF administration. This study has focused on the activation of NPCs in SCI, but much still remains to be investigated in terms of the intricate interplay of cytokines and factors, and the differentiation of NPCs also requires further investigation.
